# Supplementary material for: Oligomerisation of Ku from Mycobacterium tuberculosis promotes DNA synapsis
Source: Nat Commun. 2025 Nov 26;16:10568. doi: 10.1038/s41467-025-65609-y (PMC12658125; doi:10.1038/s41467-025-65609-y)
Supplement: Supplementary file 1 — Supplementary Information [file 41467_2025_65609_MOESM1_ESM.pdf]

Supplementary Figures

Oligomerisation of Ku from *Mycobacterium tuberculosis* promotes DNA synapsis

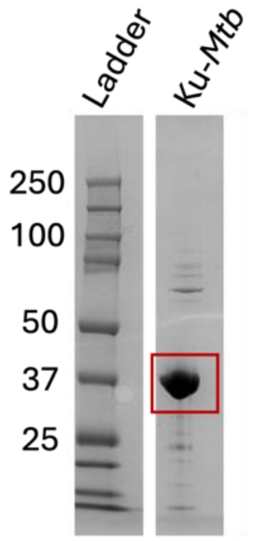

Supplementary Figure 1: 10% SDS-PAGE gel of Ku-*Mtb* after gel filtration, stained in Coomassie blue.

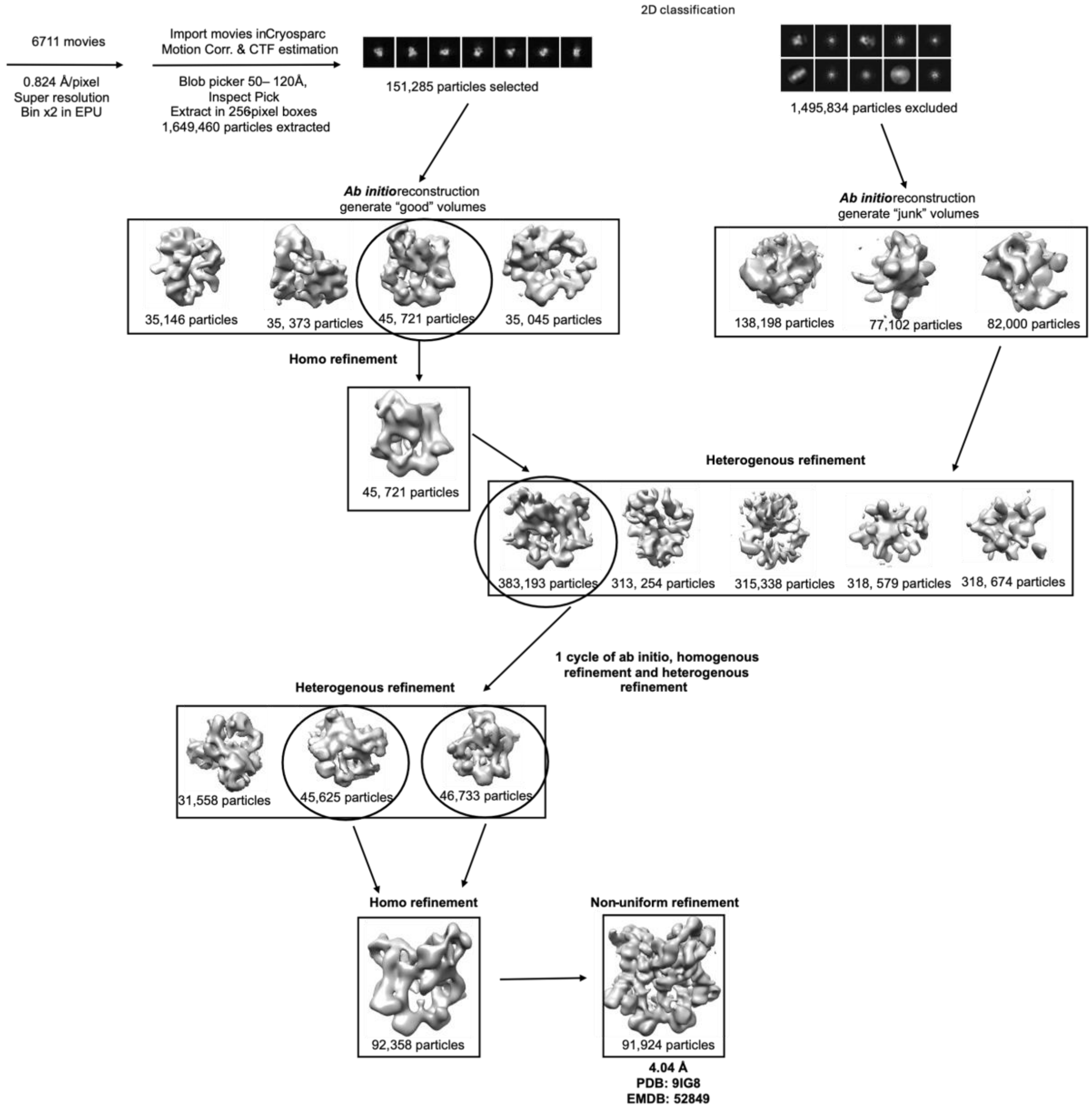

Supplementary Figure 2: Single-particle cryo-EM image processing workflow for Apo-Ku-*Mtb*. Schematic showing particle picking and processing including 2D classification, *ab initio* reconstruction and multiple heterogenous refinement cycles using CryoSPARC. The main classes generated with the corresponding number of particles are shown with black circles and the map following non-uniform refinement with resolutions for an FSC of 0.143 is given.

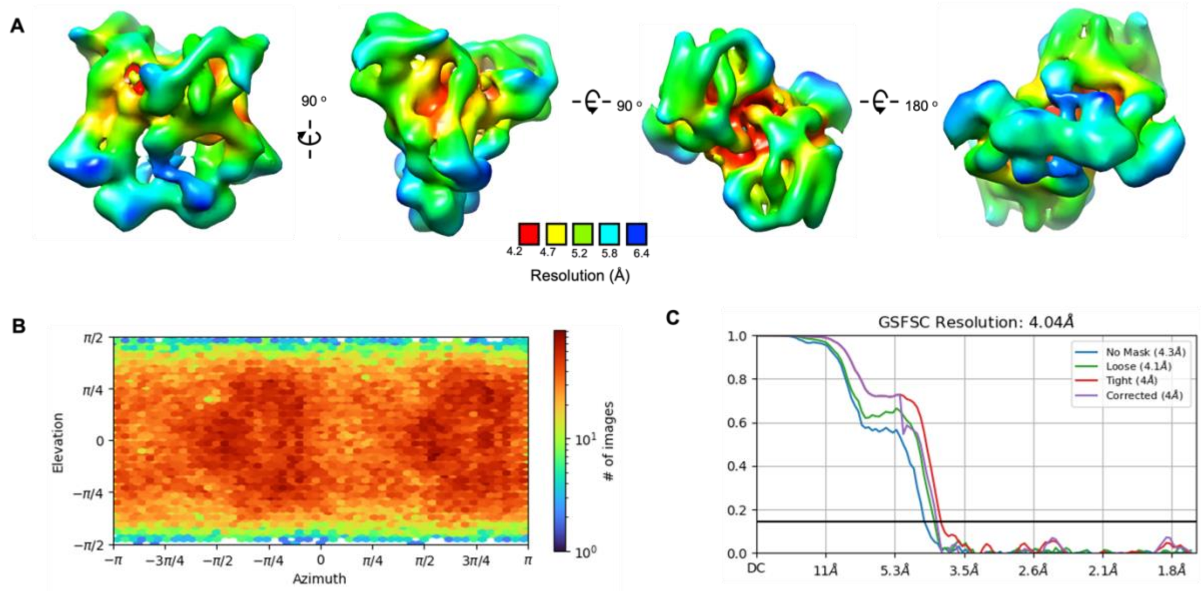

Supplementary Figure 3: Cryo-EM data of Apo-Ku-*Mtb.* A) Local resolution map of the Apo-Ku-*Mtb* cryo-EM map in four orientations with the colour key showing the corresponding resolutions. **B)** Angular distribution calculated in CryoSPARC for particle projections shown as a heat map for the consensus map. **C)** FSC resolution curves and viewing distribution plot for the consensus map.

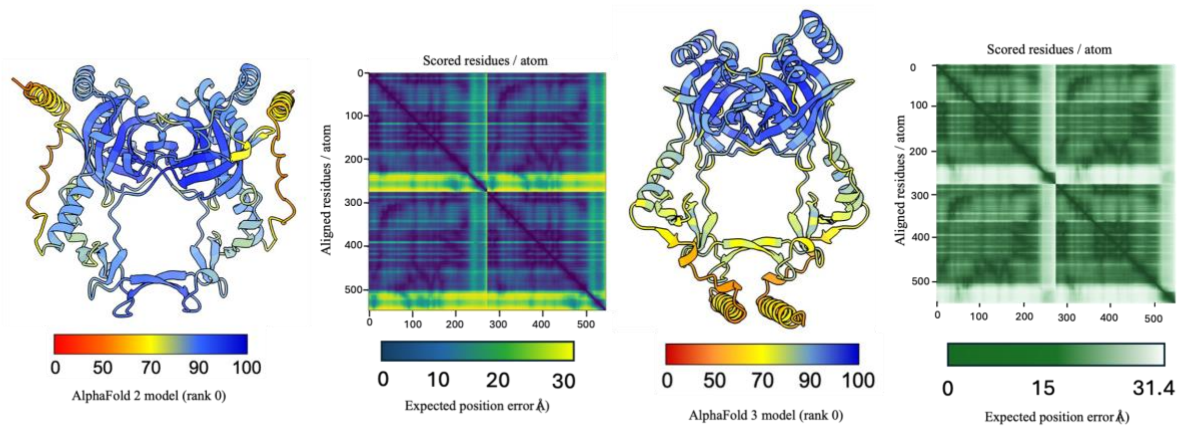

Supplementary Figure 4: AlphaFold models of Ku-*Mtb*. left, Model from AF2 coloured based on % of confidence score and its PAE plot. ipTM score = 0.81 and pTM score =0.80. Right, Model from AF3 coloured based on % of confidence score and its PAE plot. ipTM score = 0.71 and pTM score =0.73.

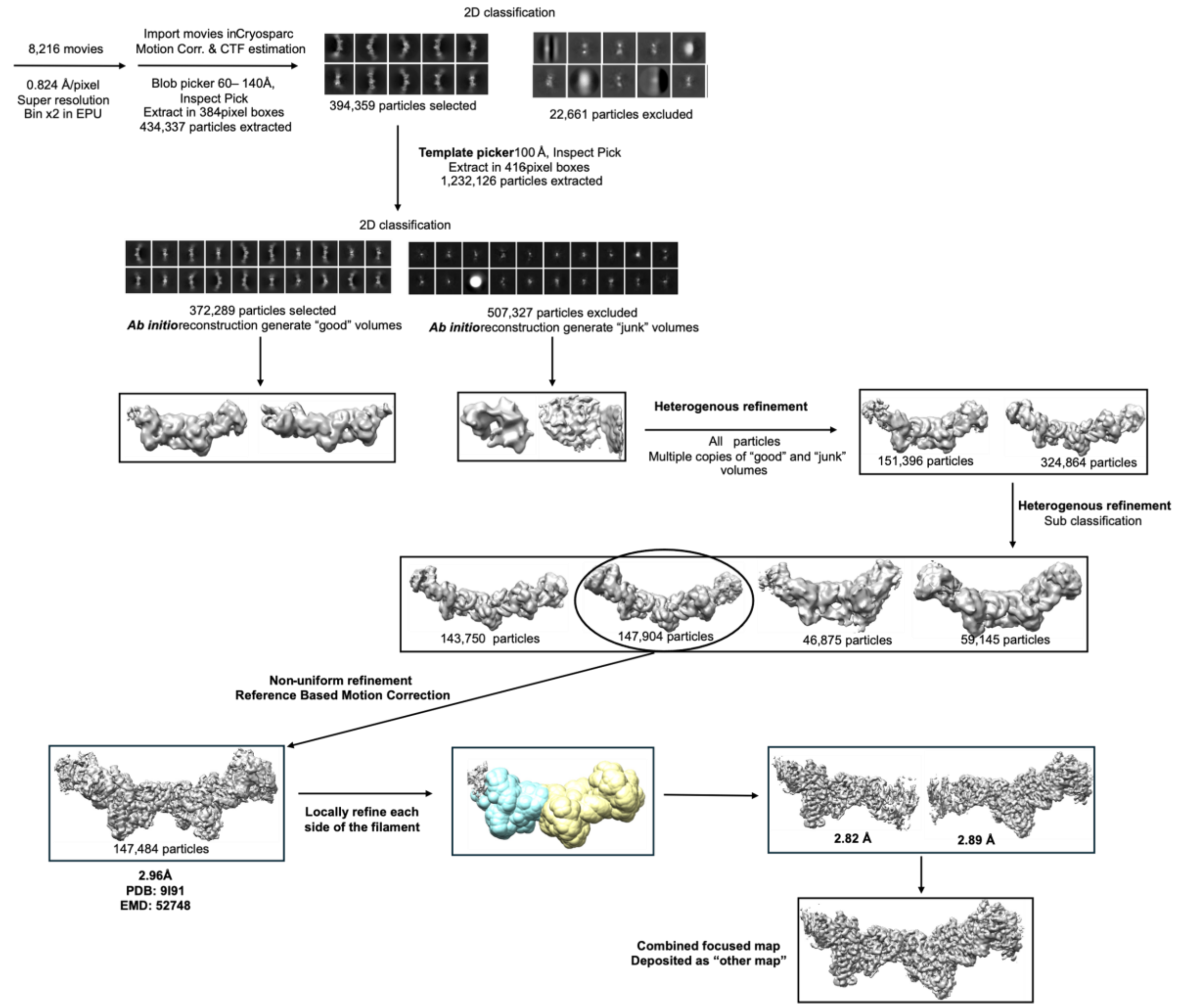

Supplementary Figure 5: Single-particle cryo-EM image processing workflow for DNA-Ku-*Mtb*. Schematic showing particle picking and processing including 2D classification, *ab initio* reconstruction and multiple heterogenous refinement cycles using CryoSPARC. The main class generated with the corresponding number of particles is shown with a black circle and the map following non-uniform refinement with resolutions for an FSC of 0.143 is given. Additional focused and composite maps are also shown.

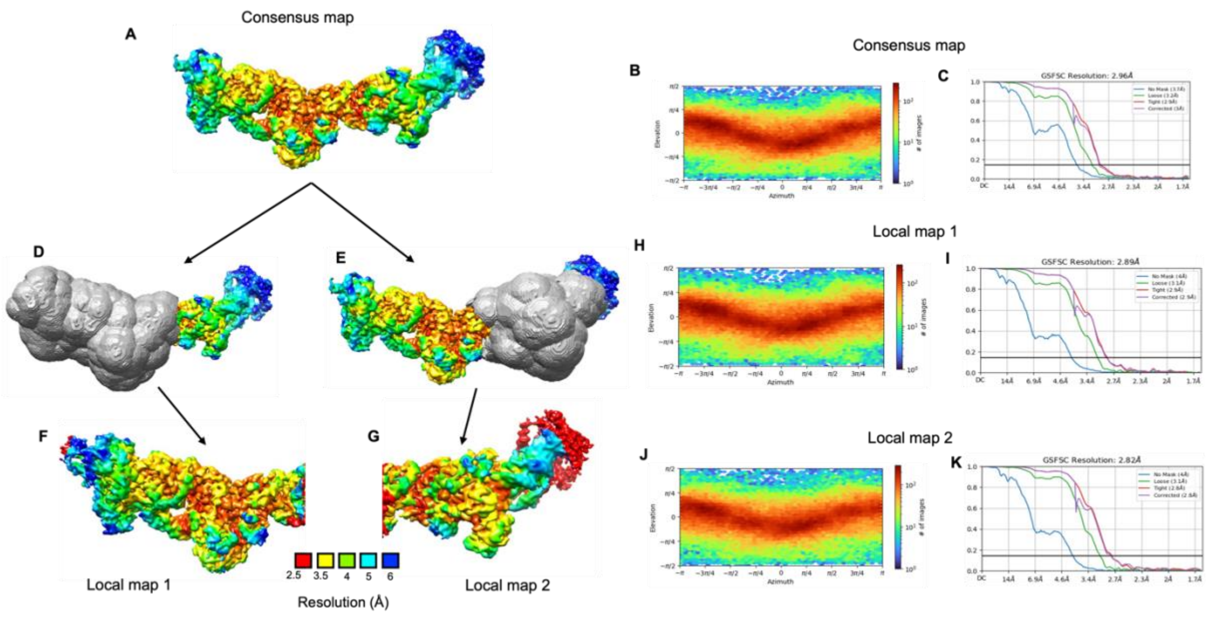

Supplementary Figure 6: Cryo-EM data of DNA-Ku-*Mtb*. A) Local resolution map of the DNA-Ku-*Mtb* consensus cryo-EM map. **B)** Angular distribution calculated in cryoSPARC for particle projections shown as a heat map for the consensus map. **C)** FSC resolution curves and viewing distribution plot for the consensus map. **D)** and **E)** DNA-Ku-*Mtb* consensus cryo-EM map with masking area. **F)** and **G)** Local resolution map of the DNA-Ku-*Mtb* locally refined map. Colours corresponding to each resolution are displayed on the specific key chart below the maps. **H)** Angular distribution calculated in cryoSPARC for particle projections shown as a heat map for local map 1. **I)** FSC resolution curves and viewing distribution plot for the local map 1. **J)** Angular distribution calculated in cryoSPARC for particle projections shown as a heat map for local map 2. **K)** FSC resolution curves and viewing distribution plot for local map 1.

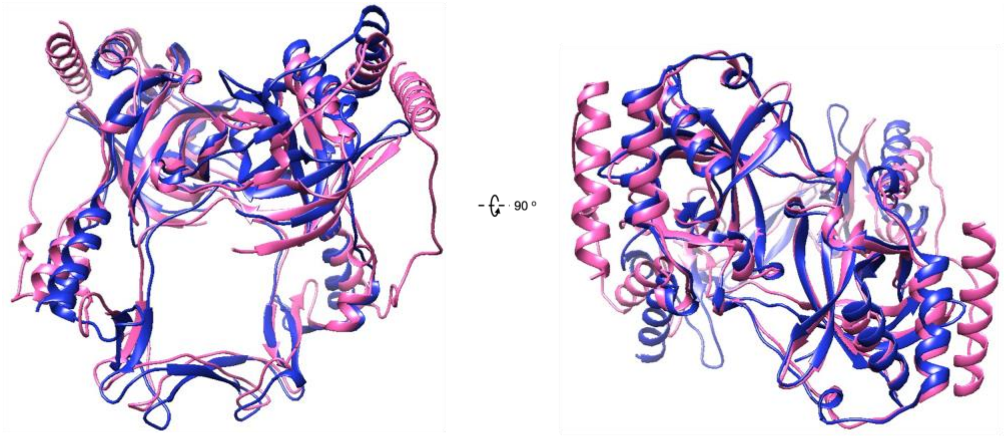

Supplementary Figure 7: Overlay of apo Ku-*Mtb* in pink and Ku-*Mtb* from Ku-*Mtb*:DNA filament in blue. RMSD 2.575.

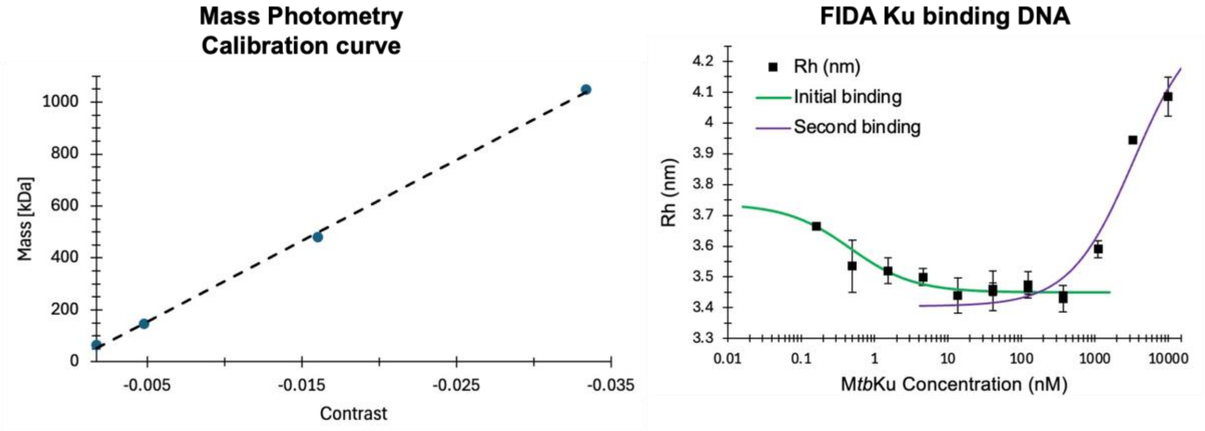

Supplementary Figure 8: Mass photometry and FIDA analysis. Left, Mass-to-contrast calibration curve. Unstained protein standard (NativeMark^{TM} , Invitrogen by Thermo Fisher Scientific) of known mass (66, 146, 480, 1048 kDa) was used to plot their mass with their contrast. The black dash line is the fit to the data according to y=ax+b, with a=-31118 and b= 2E-05, R^2 = 0.9989, max mass error: 20.6%. Right, FIDA 1 experiment of Ku-*Mtb* binding to DNA.

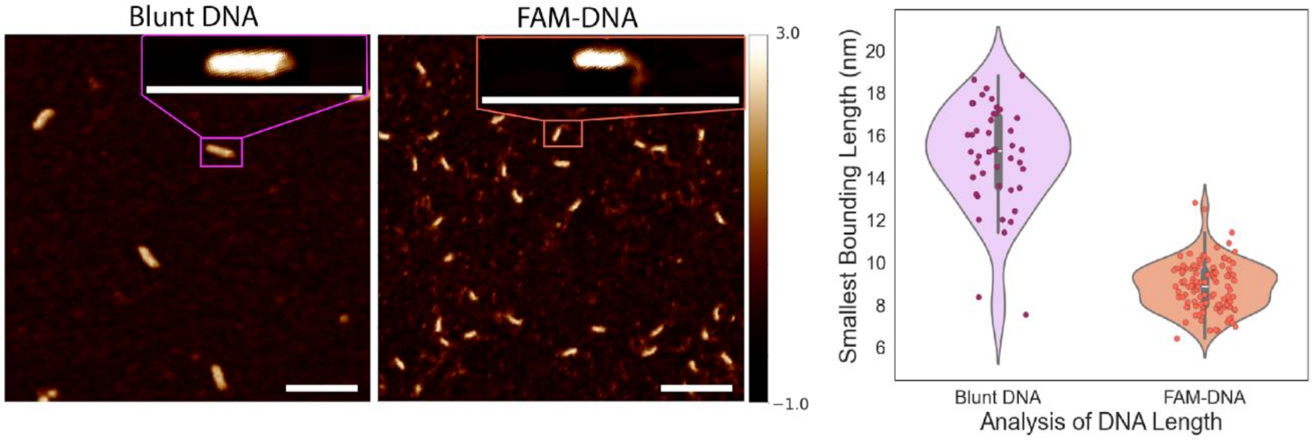

Supplementary Figure 9: Analysis of DNA oligonucleotides by AFM. Left, Representative AFM images of 50 bp blunt DNA constructs and 30 bp FAM-DNA constructs with a 20 nt single-stranded overhang. The single-stranded region is visible as lower regions connected to the molecule. The DNA constructs were imaged in liquid, immobilised using Ku buffer (75 mM NaCl, 5 mM $MgCl_2$, 20 mM TRIS pH 7.4), and imaged in 3 mM $NiCl_2$ pH 7.4. Z-scale: -1 to +3 nm main image, 0 to +2 nm inset; scale bars: 50 µm. Right, length comparisons of the double stranded region of the DNA substrates were made using measurements of smallest bounding length (nm), generated through the automated Python pipeline Topostats. Each point is representative of a single molecule. A significant reduction in the smallest bounding length was observed between blunt DNA and FAM-DNA (P-value = 2.71×10^{-19}). Number of molecules: Blunt DNA (n = 44, mean = 15 nm, SEM = 0.4) and FAM-DNA (n = 112, mean = 9 nm, SEM = 0.1).

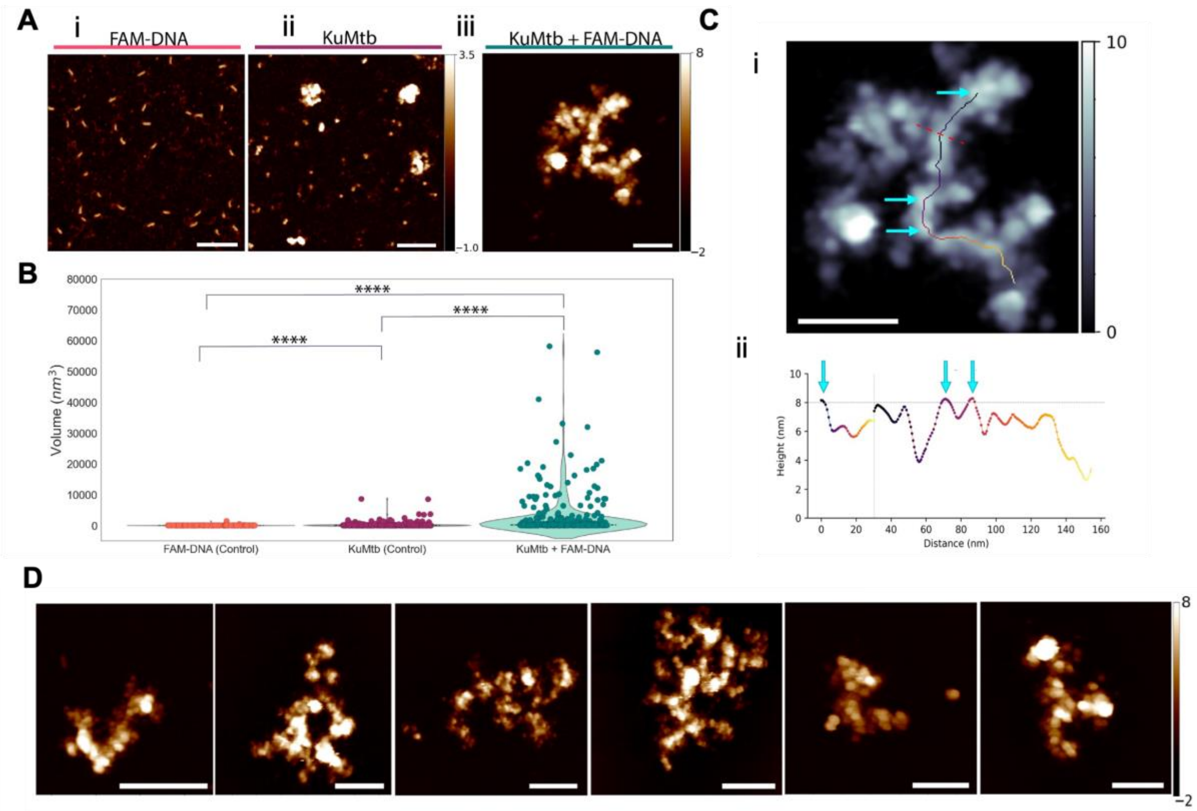

Supplementary Figure 10: AFM analysis of DNA binding to Ku-*Mtb*. A-D) Analysis of changes in Ku-*Mtb* volume following DNA binding, determined using AFM. **A)** Representative AFM images of unbound FAM-DNA (i) and Ku-*Mtb* (ii), and Ku-*Mtb* co-incubated with FAM-DNA (iii). Z-scale: 0 to +2 for A(i) and A(ii), and -2 to +8 for A(iii). **B)** The volume of each molecule was quantified using measurements of total volume (nm^3), generated using the automated Python pipeline Topostats. Each point is representative of a single molecule. There is a significant increase in Ku-*Mtb* volume in the presence of DNA Significance tested using Krustal-Wallis tests (****, P-value < 0.0001). FAM-DNA (n = 174, mean = 63 nm^3, SEM = 8.7), Ku-*Mtb* (n = 397, mean = 231 nm^3, SEM = 36.6), Ku-*Mtb* + FAM-DNA (n = 349, mean = 2466 nm^3, SEM = 358.8). **C)** An AFM image of (i) a filament-like Ku-*Mtb*-DNA complex and (ii) height profile plot of the same image, traced using the automated Python pipeline TopoStats. The coloured partial trace is comprised of 2 segments, separated by a red dashed line. Red arrows indicate the highest peaks of the height trace at 8 nm above the background. **D**) AFM images showing variation in large complexes formed after co-incubating Ku-*Mtb* with DNA. Z-scale: -2 to +8. All samples were imaged in liquid, immobilised using Ku buffer (75 mM NaCl, 5 mM $MgCl_2$, 20 mM TRIS pH 7.4), and imaged in 3 mM $NiCl_2$.Scale bars: 50 µm.

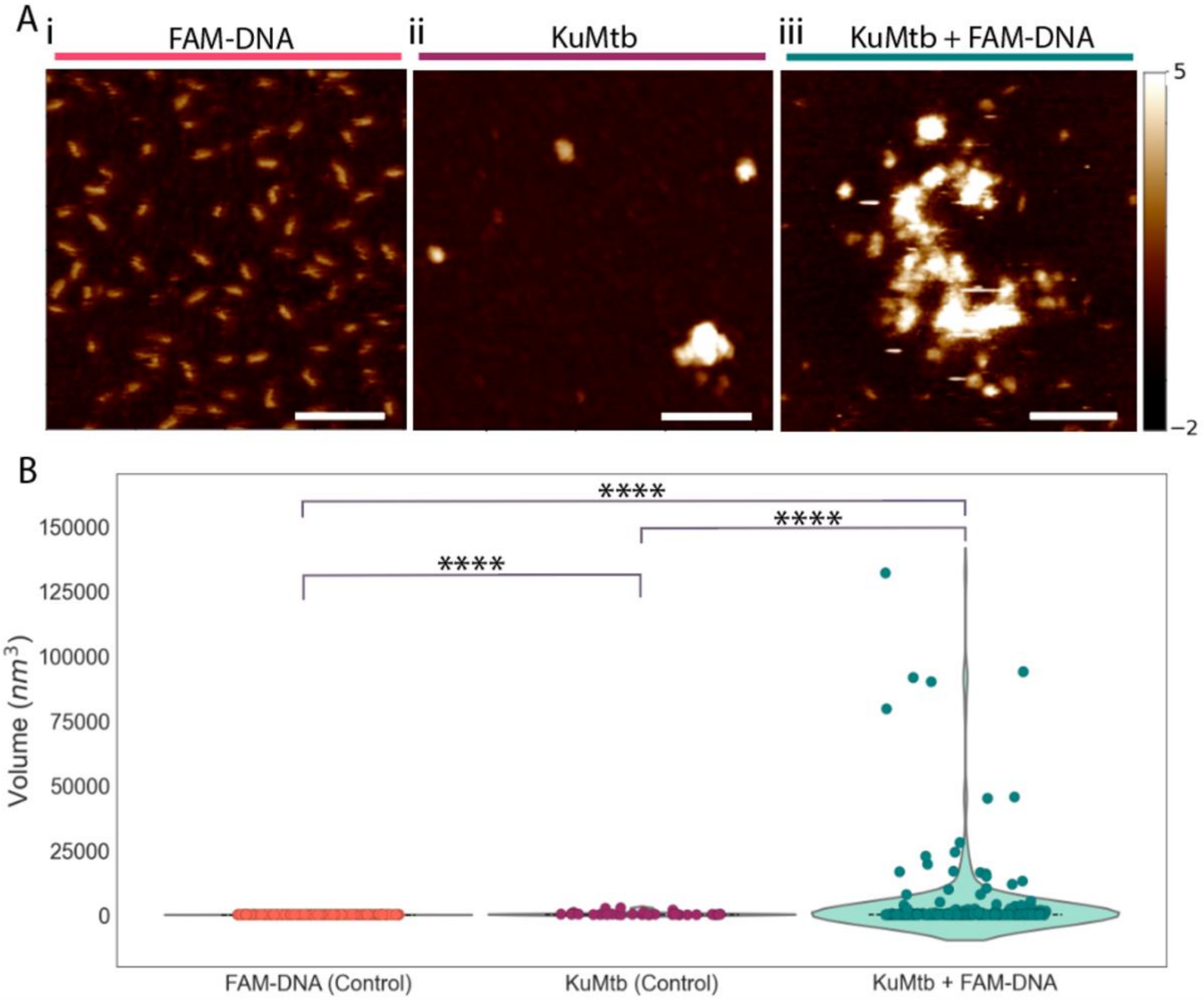

Supplementary Figure 11: Analysis of changes in Ku-*Mtb* volume following DNA binding, determined using AFM and APS immobilisation. A) Representative AFM images of unbound FAM-DNA (i) and Ku*Mtb* (ii), and Ku*Mtb* co-incubated with FAM-DNA (iii) are shown. The samples were imaged in liquid, immobilised and imaged in a buffer containing 75 mM NaCl, 20 mM TRIS pH 7.4. Z-scale: -2 to +5; scale bars:50 µm. **B)** Total volume of molecules in each condition were compared using measurements of total volume (nm^3), generated using the automated Python pipeline TopoStats. Each point is representative of a single molecule. There is a significant increase in Ku*Mtb* volume in the presence of DNA Significance tested using Krustal-Wallis tests (****, P-value < 0.0001). FAM-DNA (n = 320, mean = 88 nm^3, SEM = 2), Ku*Mtb* (n = 33, mean= 447 nm^3, SEM = 118), Ku*Mtb* + FAM-DNA (n = 253, mean = 3580 nm^3, SEM = 922).

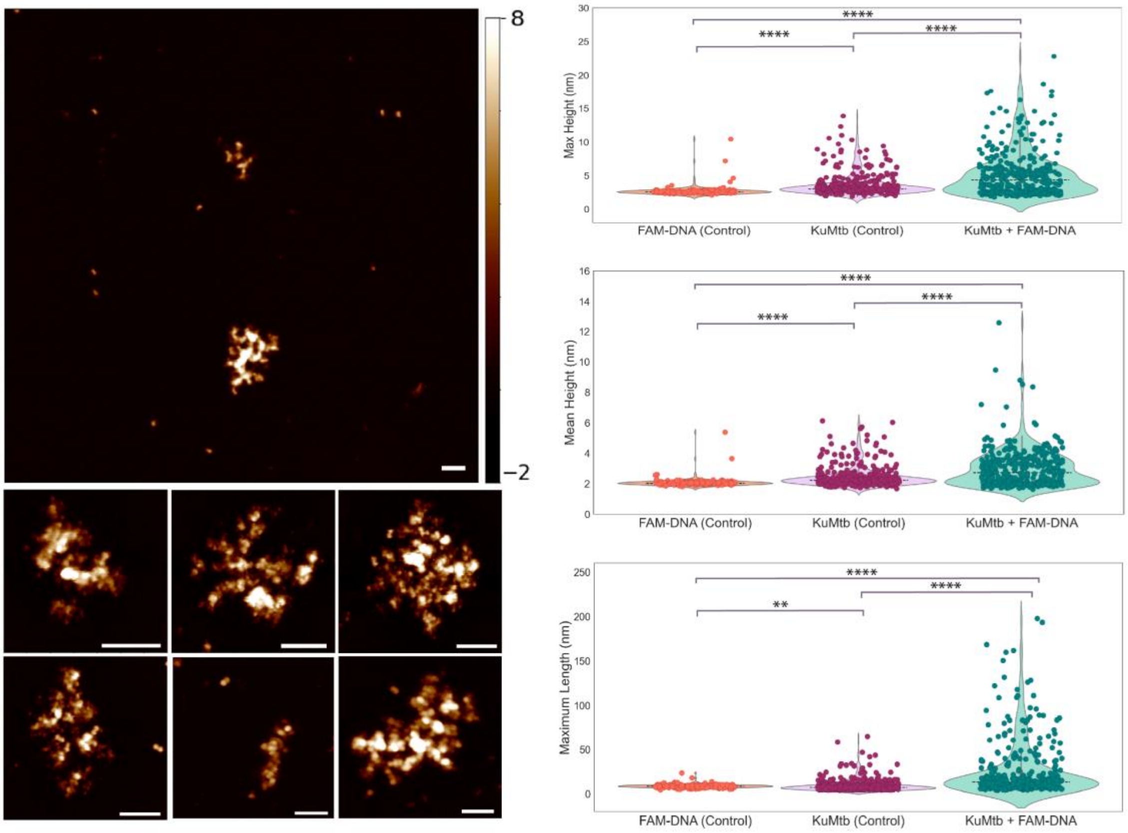

Supplementary Figure 12: AFM shows changes in Ku-*Mtb* conformation following DNA binding, quantified by changes in complex size and height of Ku-*Mtb* with DNA. Top left zoomed out image showing two large filamentous complexes and multiple individual proteins. Bottom left, zoomed in images of various filamentous complexes observed, varying in size and shape. All complexes were immobilised in 75 mM NaCl, 5 mM $MgCl_2$, 20 mM TRIS pH 7.4, and imaged in 3 mM $NiCl_2$ pH 7.4. Z-scale: -2 to +8 nm; scale bar 50 µm. Right, measurements of maximum height (top), mean height (middle) and maximum length (bottom) were generated using the automated Python pipeline TopoStats. Each point is representative of a single molecule. There is a significant increase in Ku*Mtb* mean height, maximum height and maximum length in the presence of DNA. Maximum height: FAM-DNA (n = 172, mean = 2.7 nm, SEM = 0.06), Ku*Mtb* (n = 397, mean= 3.6 nm, SEM = 0.08), Ku*Mtb* + FAM-DNA (n = 349, mean = 5.2 nm, SEM = 0.2). Mean height: FAM-DNA (n = 172, mean = 2.1 nm, SEM = 0.02), Ku*Mtb* (n = 397, mean= 2.4 nm, SEM = 0.03), Ku*Mtb* + FAM-DNA (n = 349, mean = 3.0 nm, SEM = 0.1). Maximum length: FAM-DNA (n = 172, mean = 8.6 nm, SEM = 0.2), Ku*Mtb* (n = 397, mean= 9.3 nm, SEM = 0.4), Ku*Mtb* + FAM-DNA (n = 349, mean = 25.8 nm, SEM = 1.7). Significance tested using Krustal-Wallis tests (****, P-value < 0.0001).

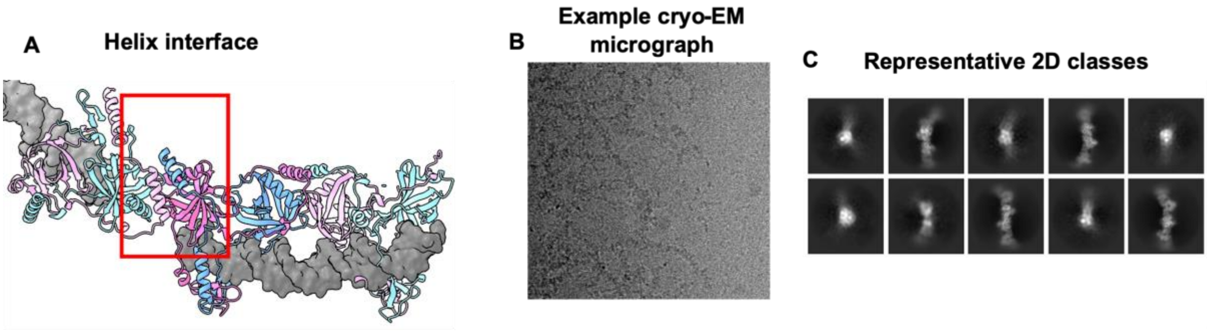

Supplementary Figure 13: Cryo-EM of disrupting the helix Ku-Ku protein interface S201A/Q197A. A) Structural model showing the helix interface. **B)** Example cryo-EM micrograph showing the filaments still forming. **C)** Representative 2D classes showing the filament formation.

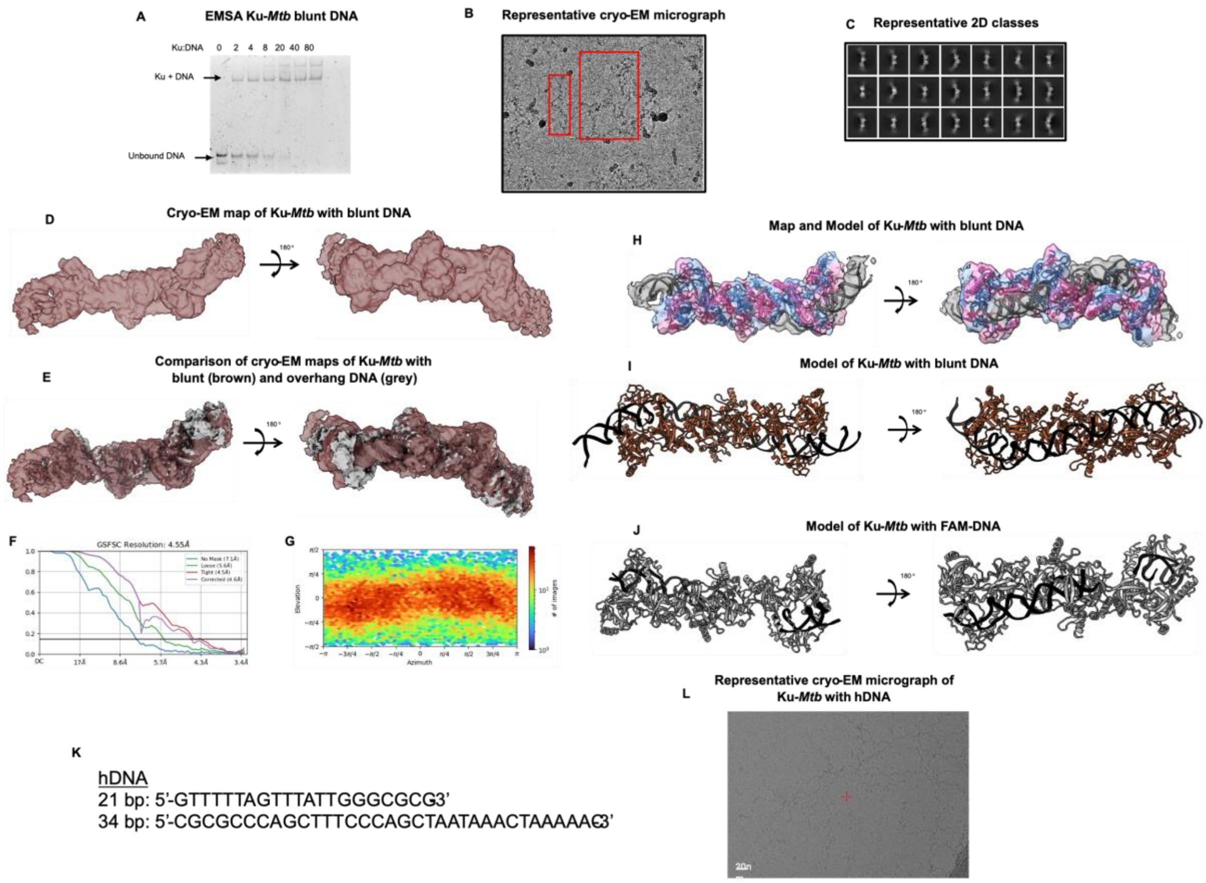

Supplementary Figure 14: Cryo-EM of Ku-*Mtb* with Blunt and hairpin DNA. A) EMSA gel of DNA with increasing ratio of Ku protein. **B)** Representative cryo-EM micrograph showing filaments of Ku-*Mtb*, with filaments highlighted with a red box. **C)** Representative

2D class averages. **D)** Cryo-EM map of Ku-*Mtb* with blunt DNA in brown. **E)** Overlaid cryo-EM maps of Ku-*Mtb* with FAM-DNA in grey with blunt DNA in brown. **F)** FSC resolution curves and viewing distribution plot of the map in D). **G)** Angular distribution calculated in cryoSPARC for particle projections shown as a heat map for the map in D). **H)** Map and model of Ku-*Mtb* with blunt DNA. **I)** model of Ku-*Mtb* with blunt DNA. **J)** model of Ku-*Mtb* with overhang DNA**. K)** DNA sequence of hairpin DNA. **L)** Representative cryo-EM micrograph of Ku-*Mtb* with hairpin DNA.

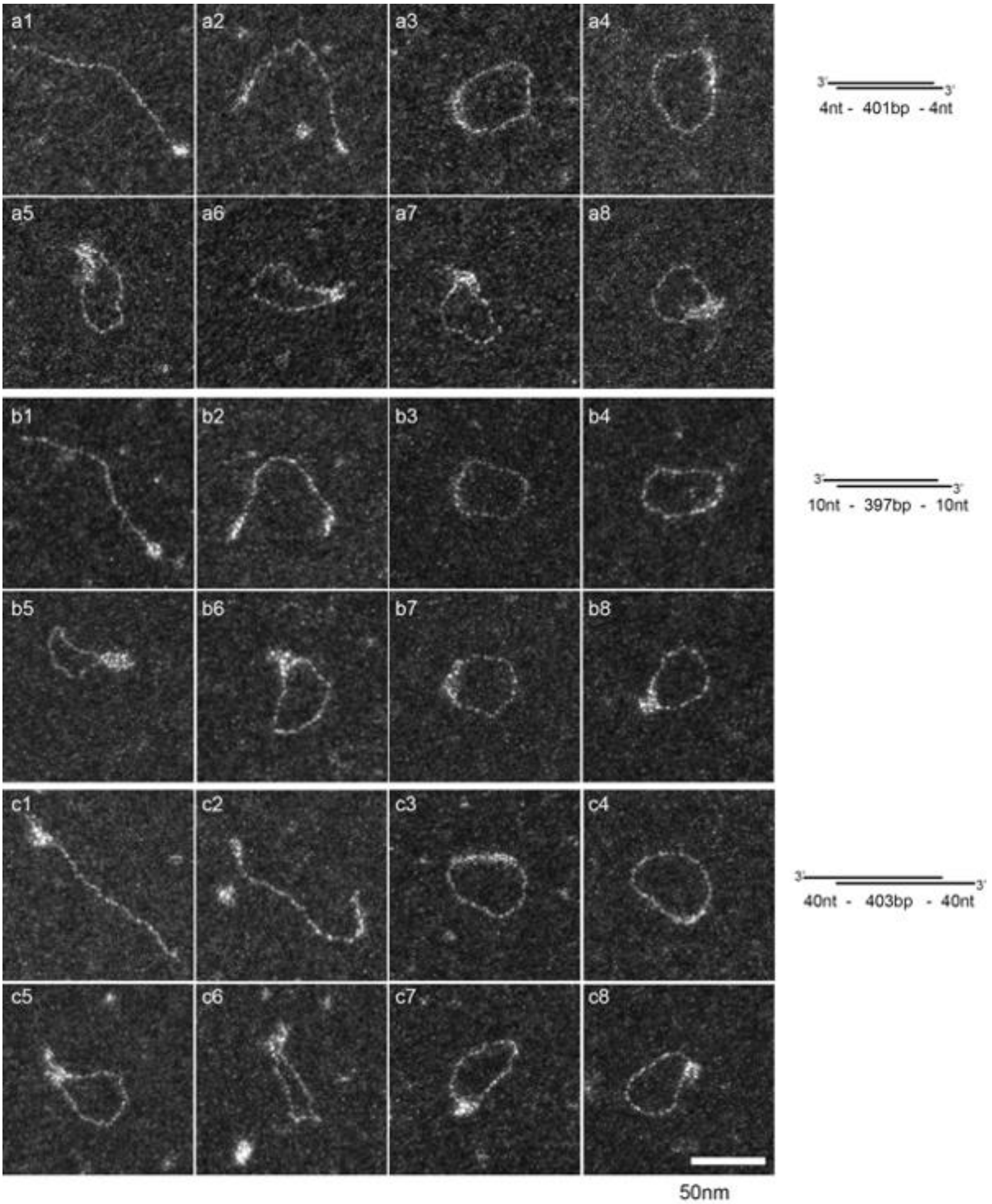

Supplementary Figure 15: Electron micrographs of DNA - Ku *Mtb* complexes obtained at 200 nM Ku with 401ds-4 ss 3’ overhang (a), 397 ds-10 ss 3’ overhang (b) and 403ds-40 ss 3’ overhang (c). The samples were analyzed in positive staining and darkfield imaging mode. This panel show Ku-DNA complexes at one end (a1,b1,c1) or at two ends (a2, b2, c2) and DNA circularization mediated by Ku *Mtb* (a3-a8, b3-b8, c3-c8). These circularization events result from by end-to -end joining. (a3-4, b3-4, c3-4), bridging B1 where the two filaments are in the

same orientation (a5-6, b5-, c5-6). or bridging B2 where the two filaments are in opposite orientation (a7-8, b-8, c7-8). The bars correspond to 50 nm.

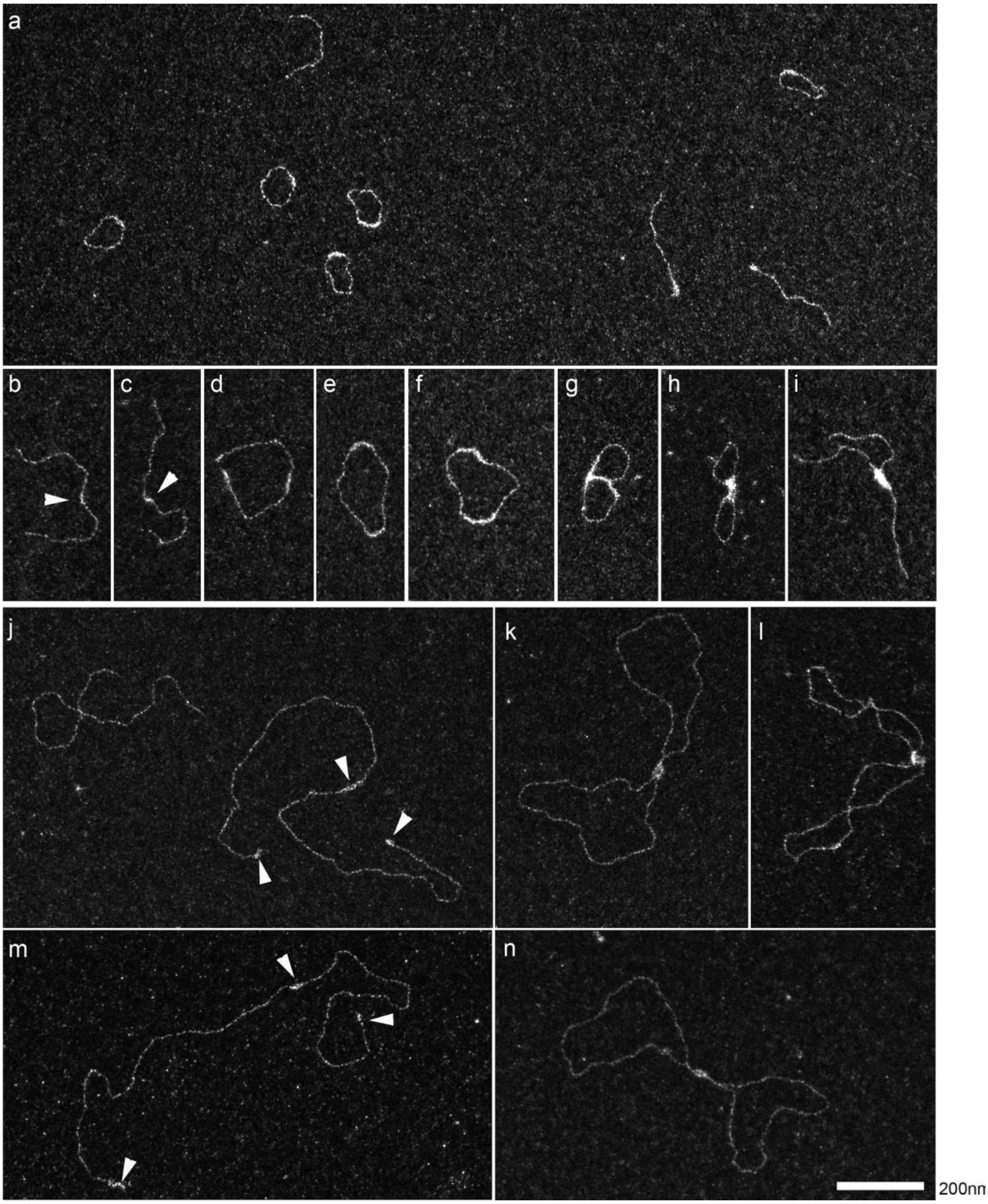

Supplementary Figure 16: Electron micrographs of singular dimers DNA – Ku-*Mtb* complexes obtained at 200 nM Ku with 401 bp (a-i) and 1440 bp (j-n) DNA fragments. (a) shows a field of various monomeric complexes, from linear to circularized complexes. (b-d) show linear dimers mediated by Ku-*Mtb* and (e-f) show dimers circularized by end-to-end

joining. (g-h) show bridging events. (i) show trimer configuration combined end to end and bridging events . (j,m) dimerization of linear 1440 bp fragment and (k,l,n) show circularization of 1440 bp dimers. The bar corresponds to 200 nm.

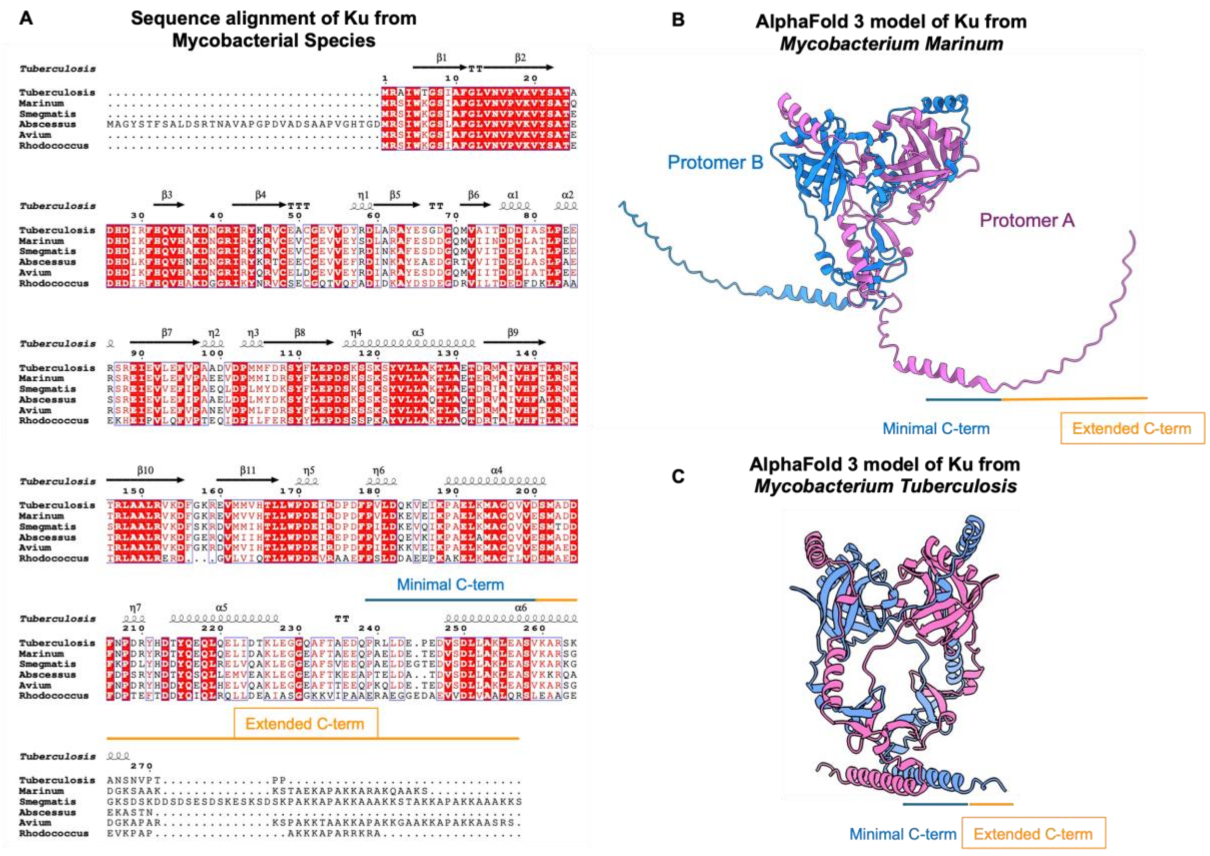

Supplementary Figure 17: Comparison of Ku from mycobacterial species. A) Sequence alignment of Ku from *Mtb* with other mycobacterial species. Secondary structure for Ku-*Mtb*

is shown above the sequence as helices of sheets and numbered. The minimal C-terminal length is shown as a dashed blue line and the extended C-terminus as orange. **B)** AlphaFold 3 model of Ku from *Mycobacterium Marinum*, which has the longest C-terminus. Protomer A in pink, protomer B in blue and the C-terminal minima and extension are labelled. **C)** AlphaFold 3 model of Ku from *Mycobacterium Tuberculosis.* Protomer A in pink, protomer B in blue and the C-terminal minima and extension are labelled.

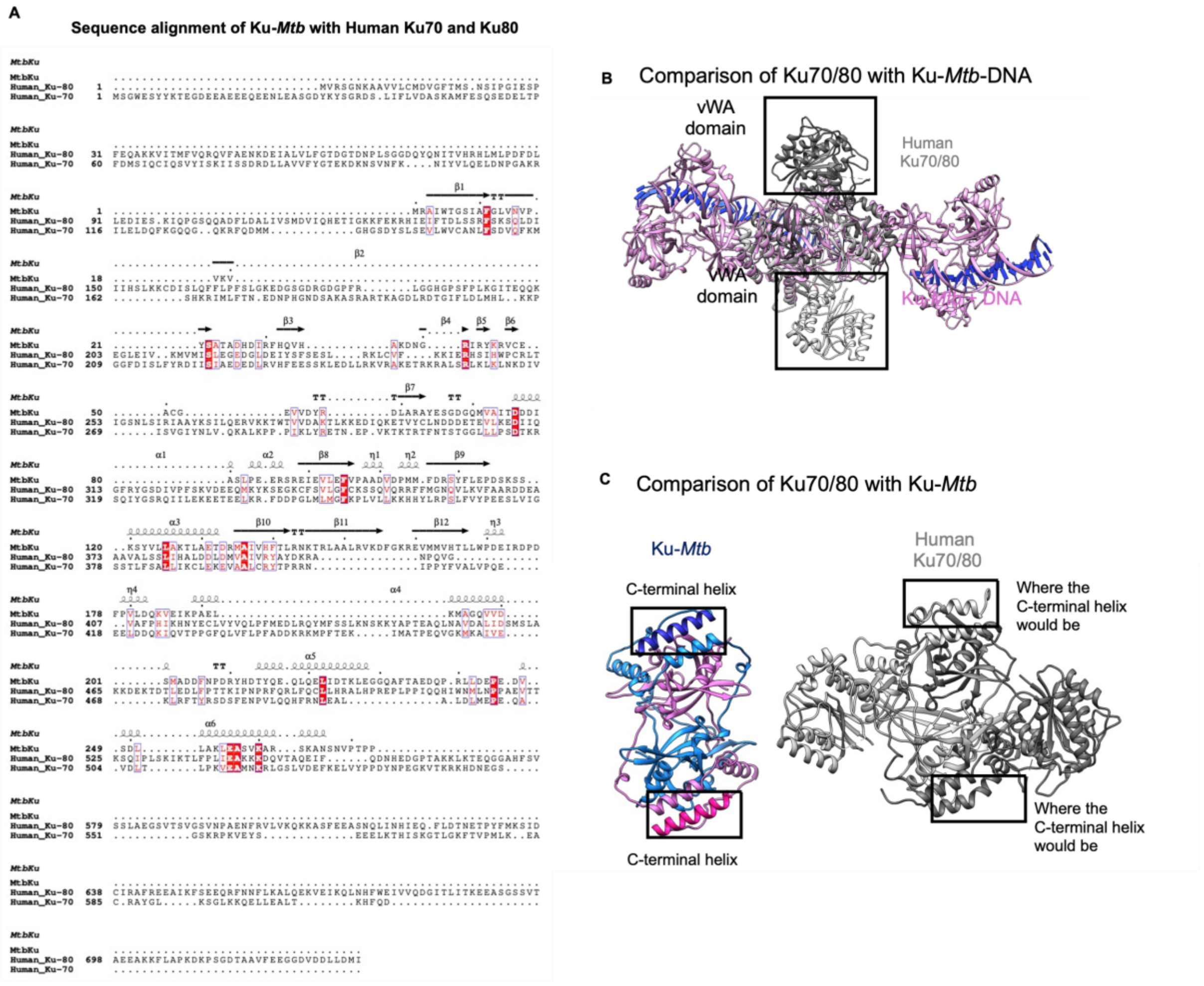

Supplementary Figure 18: Comparison of Ku from Mycobacterium Tuberculosis with Ku from Human. A) Sequence alignment of Ku from *Mtb* with human Ku. Secondary structure for Ku-*Mtb* is shown above the sequence as helices of sheets and numbered. **B)** Overlaid of DNA-Ku-*Mtb* filament structure in pink with Ku70/80 human structure (PDB: 1JEY) in grey. **C)** Comparison of Ku-*Mtb* structure with Ku70/80 human structure (PDB: 1JEY) in grey**.** For Apo-Ku-*Mtb,* protomer A is coloured in pink, with the C-terminal linker and α-helix coloured in bright pink, protomer B is coloured in blue with the C-terminal linker and α-helix coloured in dark blue.

Supplementary Table 1: Cryo-EM data collection and refinement statistics

	Apo-Ku-*Mtb*<br><br>PDB: 9IG8<br>EMD: 52849	Ku-*Mtb*-DNA<br><br>PDB: 9I91<br>EMD: 52748
**Data collection and processing**		
Detector	Gatan K3	Gatan K3
Magnification	105k	105k
Energy filter slit width (eV)	20	20
Voltage (kV)	300	300
Flux on detector (e/pix/sec)	16.2	16.6
Electron exposure on sample (e–/Å^2)	0.99	0.99
Target defocus range (µm)	-2.3 to -0.8	-2.2 to -0.7
Calibrated pixel size (Å)	0.824	0.824
Symmetry imposed	C2	C1
Extraction box size (pixels)	256	416
Initial particle images (no.)	151,285	1,232,126
Final particle images (no.)	91,924	147,484
**Refinement**		
Map resolution at FSC=0.143 (Å)*	4.04	2.96
Model composition		
Non-hydrogen atoms	4251	12011
Protein residues	534	1374
Nucleotides		88
B factor (Å^2)		
Protein	241.92	131.87
DNA		167.43
R.m.s deviations		
Bond lengths (Å)	0.003	0.004
Bond angles (°)	0.836	167.43
Validation		
Molprobity score	2.19	2.23
Clashscore	15.66	10.45
Poor rotamers (%)	2.17	3.36
Ramachandran plot		
Favored (%)	96.42	95.74
Allowed (%)	3.40	4.26
Disallowed (%)	0.19	0

Supplementary Table 2: Analysis of Ku-DNA complexes at 200 nM Ku in terms of percentages of complexes, linear or circularized, of covered DNA sizes by Ku, and of events promoting circularization, ie end-to-end or bridging.

DNA fragments		*401pb blunt n=282*	*401pb +4n 3' n=258*	*397 pb+10n 3' n=200*	*403pb+40n 3' n=317*
% of complexes		*85 (n=241)*	*84 (n=216)*	*79 (n=159)*	*84 (n=267)*
% of linear		*12*	*16*	*6*	*40*
	% of one end linked	*70*	*40*	*45*	*40*
	% of two ends linked	*30*	*60*	*55*	*60*
% of circular		*88*	*84 (n=182)*	*94 (n=149)*	*60 (n=151)*
Circular	*% of End to end*	*45*	*28*	*42*	*25*
	% of Bridges (b1/b2)	*55*	*72 (32/56)*	*58 (17/83)*	*75 (39/61)*
Covered length by Ku	*Linear*	*16,6 nm +/-6 (50 bp +/-17)*	*17,7 nm +/-6 (52 bp +/- 17)*	*20,2 nm +/-7 (58 bp +/- 20)*	*17,7nm +/-6 (52 bp +/-17)*
	circular	*30.9 nm +/-8 (91 pb +/-23)*	*39 nm +/-10 (114 bp +/-29)*	*40,4 nm+/- 14 116 bp +/-40*	*35 nm +/-8 (102 bp +/-23)*

Supplementary Table 3: DNA substrates for positive staining EM experiments

**Blunt DNA 1440ds fragment**		
Primer 1 : 5'GGATCTCAACAGCGGTAA3'	Primer 2 5'CGACGCTCAAGTCAGAGG3'	

```
5'GGATCTCAACAGCGGTAAgatccttgagagttttcgccccgaagaacgttttccaatgatgagcacttttaaagttctgctatgtg
3'CCTAGAGTTGTCGCCATTctaggaactctcaaaagcggggcttcttgcaaaaggttactactcgtgaaaatttcaagacgatacac

  gcgcggtattatcccgtgttgacgccgggcaagagcaactcggtcgccgcatacactattctcagaatgacttggttgagtactcacc
  cgcgccataatagggcacaactgcggcccgttctcgttgagccagcggcgtatgtgataagagtcttactgaaccaactcatgagtgg

  agtcacagaaaagcatcttacggatggcatgacagtaagagaattatgcagtgctgccataaccatgagtgataacactgcggccaac
  tcagtgtcttttcgtagaatgcctaccgtactgtcattctcttaatacgtcacgacggtattggtactcactattgtgacgccggttg

  ttacttctgacaacgatcggaggaccgaaggagctaaccgcttttttgcacaacatgggggatcatgtaactcgccttgatcgttggg
  aatgaagactgttgctagcctcctggcttcctcgattggcgaaaaaacgtgttgtaccccctagtacattgagcggaactagcaaccc

  aaccggagctgaatgaagccataccaaacgacgagcgtgacaccacgatgcctgcagcaatggcaacaacgttgcgcaaactattaac
  ttggcctcgacttacttcggtatggtttgctgctcgcactgtggtgctacggacgtcgttaccgttgttgcaacgcgtttgataattg

  tggcgaactacttactctagcttcccggcaacaattaatagactggatggaggcggataaagttgcaggaccacttctgcgctcggcc
  accgcttgatgaatgagatcgaagggccgttgttaattatctgacctacctccgcctatttcaacgtcctggtgaagacgcgagccgg

  cttccggctggctggtttattgctgataaatctggagccggtgagcgtgggtctcgcggtatcattgcagcactggggccagatggta
  gaaggccgaccgaccaaataacgactatttagacctcggccactcgcacccagagcgccatagtaacgtcgtgaccccggtctaccat
```

```
agccctcccgtatcgtagttatctacacgacggggagtcaggcaactatggatgaacgaaatagacagatcgctgagataggtgcctc
tcgggagggcatagcatcaatagatgtgctgcccctcagtccgttgatacctacttgctttatctgtctagcgactctatccacggag

actgattaagcattggtaactgtcagaccaagtttactcatatatactttagattgatttaaaacttcatttttaatttaaaaggatc
tgactaattcgtaaccattgacagtctggttcaaatgagtatatatgaaatctaactaaattttgaagtaaaaattaaattttcctag

taggtgaagatcctttttgataatctcatgaccaaaatcccttaacgtgagttttcgttccactgagcgtcagaccccgtagaaaaga
atccacttctaggaaaaactattagagtactggttttagggaattgcactcaaaagcaaggtgactcgcagtctggggcatcttttct

tcaaaggatcttcttgagatcctttttttctgcgcgtaatctgctgcttgcaaacaaaaaaaccaccgctaccagcggtggtttgttt
agtttcctagaagaactctaggaaaaaaagacgcgcattagacgacgaacgtttgttttttttggtggcgatggtcgccaccaaacaaa

gccggatcaagagctaccaactctttttccgaaggtaactggcttcagcagagcgcagataccaaatactgtccttctagtgtagccg
cggcctagttctcgatggttgagaaaaaggcttccattgaccgaagtcgtctcgcgtctatggtttatgacaggaagatcacatcggc

tagttaggccaccacttcaagaactctgtagcaccgcctacatacctcgctctgctaatcctgttaccagtggctgctgccagtggcg
atcaatccggtggtgaagttcttgagacatcgtggcggatgtatggagcgagacgattaggacaatggtcaccgacgacggtcaccgc

ataagtcgtgtcttaccgggttggactcaagacgatagttaccggataaggcgcagcggtcgggctgaacggggggttcgtgcacaca
tattcagcacagaatggcccaacctgagttctgctatcaatggcctattccgcgtcgccagcccgacttgccccccaagcacgtgtgt

gcccagcttggagcgaacgacctacaccgaactgagatacctacagcgtgagctatgagaaagcgccacgcttcccgaagggagaaag
cgggtcgaacctcgcttgctggatgtggcttgactctatggatgtcgcactcgatactctttcgcggtgcgaagggcttccctctttc

gcggacaggtatccggtaagcggcagggtcggaacaggagagcgcacgagggagcttccagggggaaacgcctggtatctttatagtc
cgcctgtccataggccattcgccgtcccagccttgtcctctcgcgtgctccctcgaaggtccccctttgcggaccatagaaatatcag
gacagcccaaagcggtGGAGACTGAACTCGCAGC

ctgtcgggtttcgccaCCTCTGACTTGAGCGTCG3’
gacagcccaaagcggtGGAGACTGAACTCGCAGC5’
```

**Blunt DNA 401ds fragment**		
Primer 1 :<br>5’ACGCTCAAGTCAGAGGTG3’	Primer 2<br>5’TCCTTCTAGTGTAGCCGTAGTT3'	

DNA 401ds

```
5’ACGCTCAAGTCAGAGGTGGcgaaacccgacaggactataaagataccaggcgtttccccctggaagctccctcgtgcgctctcctg
3’TGCGAGTTCAGTCTCCACCgctttgggctgtcctgatatttctatggtccgcaaagggggaccttcgagggagcacgcgagaggac

ttccgaccctgccgcttaccggatacctgtccgcctttctcccttcgggaagcgtggcgctttctcatagctcacgctgtaggtatct
aaggctgggacggcgaatggcctatggacaggcggaaagagggaagcccttcgcaccgcgaaagagtatcgagtgcgacatccataga

cagttcggtgtaggtcgttcgctccaagctgggctgtgtgcacgaaccccccgttcagcccgaccgctgcgccttatccggtaactat
gtcaagccacatccagcaagcgaggttcgacccgacacacgtgcttggggggcaagtcgggctggcgacgcggaataggccattgata

cgtcttgagtccaacccggtaagacacgacttatcgccactggcagcagccactggtaacaggattagcagagcgaggtatgtaggcg
gcagaactcaggttgggccattctgtgctgaatagcggtgaccgtcgtcggtgaccattgtcctaatcgtctcgctccatacatccgc

gtgctacagagttcttgaagtggtggcctAACTACGGCTACACTAGAAGGA3’
cacgatgtctcaagaacttcaccaccggaTTGATGCCGATGTGATCTTCCT5’
```

Overhang substrats		
**DNA401ds-4ss 3’ overhang**		
Single Strand 1		
Primer 1<br>Biot 5’ACAAAAATCGACGCTCAAGT3’	Primer 2<br>5’TAGTGTAGCCGTAGTTAGGCCAC3’	

3’**TGTT**TTTAGCTGCGAGTTCAgtctccaccgctttgggctgtcctgatatttctatggtccgcaaagggggaccttcgagggagcacg
cgagaggacaaggctgggacggcgaatggcctatggacaggcggaaagagggaagcccttcgcaccgcgaaagagtatcgagtgcgaca
tccatagagtcaagccacatccagcaagcgaggttcgacccgacacacgtgcttggggggcaagtcgggctggcgacgcggaataggcc

attgatagcagaactcaggttgggccattctgtgctgaatagcggtgaccgtcgtcggtgaccattgtcctaatcgtctcgctccatac<br>atccgccacgatgtctcaagaacttcacCACCGGATTGATGCCGATGTGAT5’		
Single Strand 2		
Primer 1<br>5’AAATCGACGCTCAAGTCAGAGGT3'	Primer 2<br>Biot-5’CTTCTAGTGTAGCCGTAGTTA3'	
5’AAATCGACGCTCAAGTCAGAGGTggcgaaacccgacaggactataaagataccaggcgtttccccctggaagctccctcgtgcgct<br>ctcctgttccgaccgctccaagctgggctgtgtgcacgaaccccccgttcagcccgaccgctgcgccttatccggtaactatcgtctt<br>gagtccaacccggtaagacacgacttatcgccactggcagcagccactggtaacaggattagcagagcgaggtatgtaggcggtgcta<br>cagagttcttgaagtggtggccTAACTACGGCTACACTA**GAAG**3’		
**DNA397ds-10ss 3’ overhang**		
Single Strand 1		
Primer 1<br>Biot 5’AGCATCACAAAAATCGACGCTC3’	Primer 2<br>5’GTAGCCGTAGTTAGGCCACCACT3’	
3’**TCGTAGTGTT**TTTAGCTGCGAGttcagtctccaccgctttgggctgtcctgatatttctatggtccgcaaaggggaccttcgagg<br>Gagcacgcgagaggacaaggctgggacggcgaatggcctatggacaggcggaaagagggaagcccttcgcaccgcgaaagagtatcgag<br>tgcgacatccatagagtcaagccacatccagcaagcgaggttcgacccgacacacgtgcttgggggcaagtcgggctggcgacgcgga<br>ataggccattgatagcagaactcaggttgggccattctgtgctgaatagcggtgaccgtcgtcggtgaccattgtcctaatcgtctcgc<br>tccatacatccgccacgatgtctcaagaactTCACCACCGGATTGATGCCGATG5’		
Single Strand 2		
Primer 1<br>5’AAATCGACGCTCAAGTCAGAG3’	Primer 2<br>Biot-5’TCCTTCTAGTGTAGCCGTAGTT3’	
5’AAATCGACGCTCAAGTCAGAGgtggcgaaacccgacaggactataaagataccaggcgtttccccctggaagctccctcgtgcgctc<br>tcctgttccgaccctgccgcttaccggatacctgtccgcctttctcccttcgggaagcgtggcgctttctcatagctcacgctgtaggt<br>atctcagttcggtgtaggtcgttcgctccaagctgggctgtgtgcacgaaccccccgttcagcccgaccgctgcgccttatccggtaac<br>tatcgtcttgagtccaacccggtaagacacgacttatcgccactggcagcagccactggtaacaggattagcagagcgaggtatgtagg<br>cggtgctacagagttcttgaagtggtggcctAACTACGGCT**ACACTAGAAGGA**3’		
**DNA 403ds-40ss 3’ overhang**		
Single Strand 1		
Primer 1	Primer 2	
Biot-5’TTTCCATAGGCTCCGCCC3’	5’TCCTTCTAGTGTAGCCGTAGTT3’	
3’**AAAGGTATCCGAGGCGGGgggactgctcgtagtgtttta**gctgcgagttcagtctccaccgctttgggctgtcctgatatttct<br>Atggtccgcaaaggggaccttcgagggagcacgcgagaggacaaggctgggacggcgaatggcctatggacaggcggaaagagggaag<br>cccttcgcaccgcgaaagagtatcgagtgcgacatccatagagtcaagccacatccagcaagcgaggttcgacccgacacacgtgcttg<br>ggggcaagtcgggctggcgacgcggaataggccattgatagcagaactcaggttgggccattctgtgctgaatagcggtgaccgtcgt<br>cggtgaccattgtcctaatcgtctcgctccatacatccgccacgatgtctcaagaacttcaccaccggaTTGATGCCGATGTGATCTTC<br>CT5’		
Single Strand2-		
Primer 1	Primer 2	
5’ACGCTCAAGTCAGAGGTG	Biot-5’CGAAGGTAACTGGCTTCAGCA	
3’**GCTTCCATTGACCGAAGTCGTctcgcgtctatggtttatg**acaggaagatcacatcggcatcaatccggtggtgaagttcttgagac<br>atcgtggcggatgtatggagcgagacgattaggacaatggtcaccgacgacggtcaccgctattcagcacagaatggcccaacctgagt<br>tctgctatcaatggcctattccgcgtcgccagcccgacttgcccccaagcacgtgtgtcgggtcgaacctcgcttgctggatgtggct<br>tgactctatggatgtcgcactcgatactctttcgcggtgcgaagggcttccctctttccgcctgtccataggccattcgccgtcccagc<br>cttgtcctctcgcgtgctccctcgaaggtcccccttttgcggaccatagaaatatcaggacagcccaaagcgGTGGAGACTGAACTCGCA<br>5’		

Source Data:

Supplementary Figure 1

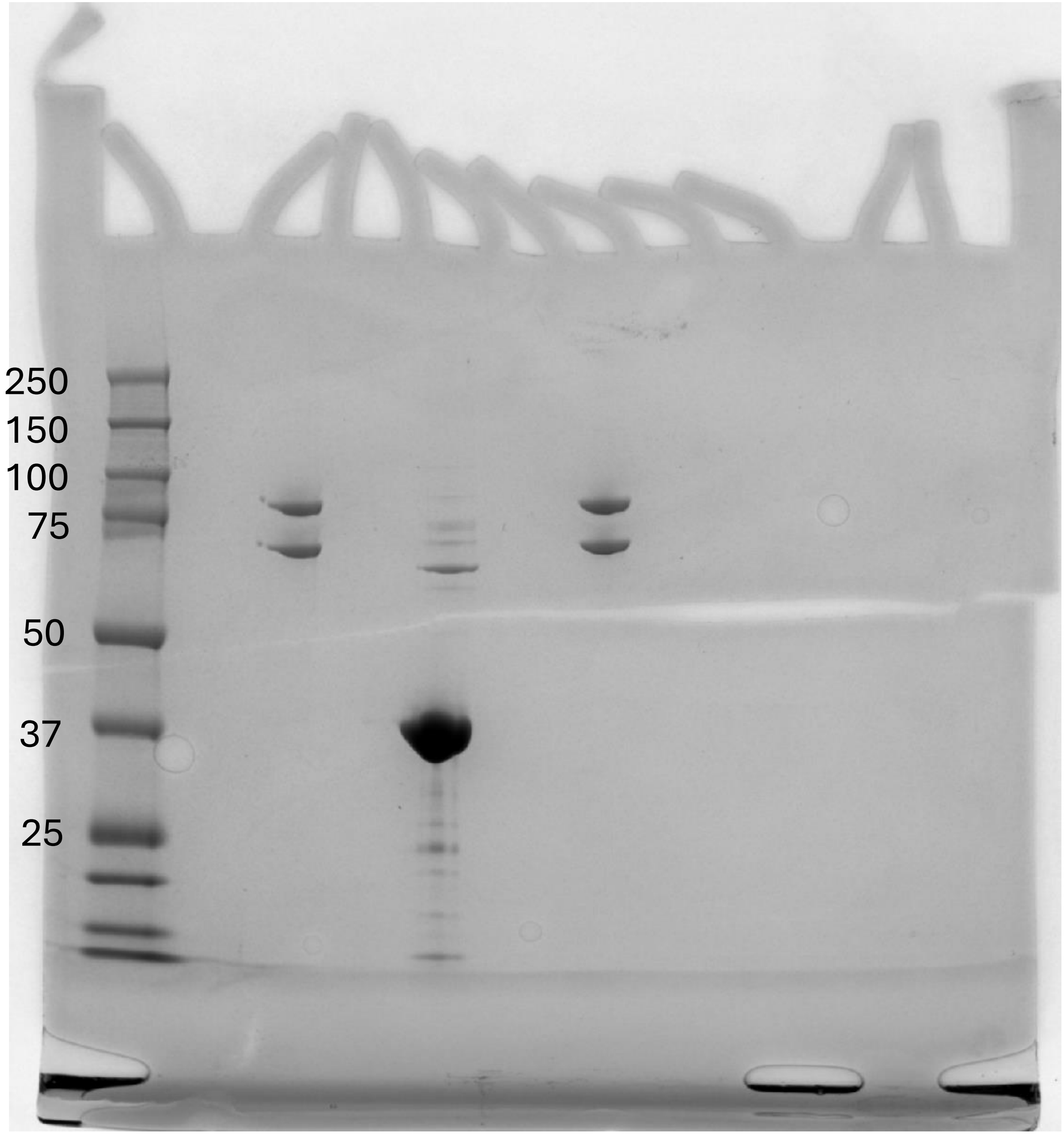

Supplementary Figure 8B

Conc, [nM]	Rh [nm]	Rh Std, Dev	Binding cur	Binding cur	Conc, [nM]	Rh [nm]	Rh Std, Dev	Binding cur	Binding cur
	Initial bind			Initial bind		Second bin			Second bin
0	3.76	0.084853	0.016	3.728857	41.1	3.455	0.06364	4.11	3.405366
0.16	3.665	0.06364	0.020143	3.726453	123	3.465	0.021213	5.174183	3.405661
0.5	3.535	0.007071	0.025358	3.723483	370	3.43	0.042426	6.513911	3.406032
1.52	3.52	0.084853	0.031924	3.719834	1111	3.59	0.028284	8.200528	3.406499
4.57	3.5	0.042426	0.04019	3.715377	3333	3.945	0.007071	10.32385	3.407086
13.7	3.44	0.028284	0.050596	3.709969	10000	4.085	0.06364	12.99696	3.407824
41.1	3.46	0.056569	0.063697	3.703467				16.36221	3.408751
123	3.475	0.021213	0.08019	3.695728				20.5988	3.409916
370	3.44	0.042426	0.100953	3.686631				25.93235	3.411378
			0.127093	3.676091				32.64689	3.413213
			0.16	3.664083				41.1	3.415512
			0.201428	3.650662				51.74183	3.418391
			0.253583	3.635977				65.13911	3.42199
			0.319242	3.62028				82.00528	3.426482
			0.401902	3.603915				103.2385	3.432075
			0.505964	3.587289				129.9696	3.439021
			0.636971	3.570838				163.622	3.447618
			0.8019	3.554979				205.988	3.458213
			1.009532	3.540071				259.3235	3.471203
			1.270925	3.526385				326.4689	3.487029
			1.6	3.51409				411	3.506162
			2.014281	3.50326				517.4183	3.529077
			2.535829	3.493883				651.3911	3.556216
			3.19242	3.485884				820.0528	3.587937
			4.019018	3.479148				1032.385	3.624445
			5.059644	3.473536				1299.696	3.665723
			6.369715	3.468902				1636.22	3.711468
			8.018996	3.465103				2059.88	3.761051
			10.09532	3.462009				2593.235	3.813517
			12.70925	3.459501				3264.689	3.867642
			16	3.457476				4110	3.922034
			20.14281	3.455847				5174.183	3.975273
			25.35829	3.454539				6513.911	4.026058
			31.9242	3.453492				8200.528	4.073325
			40.19018	3.452654				10323.85	4.11632
			50.59644	3.451985				12996.96	4.154622
			63.69715	3.451452				16362.2	4.188114
			80.18996	3.451026				20598.8	4.216926
			100.9532	3.450688				25932.35	4.241367
			127.0925	3.450418				32646.89	4.261855
			160	3.450204				41100	4.278858
			201.4281	3.450033					
			253.5829	3.449897					
			319.242	3.44979					
			401.9018	3.449704					
			505.9644	3.449636					
			636.9715	3.449582					
			801.8996	3.449539					
			1009.532	3.449504					
			1270.925	3.449477					
			1600	3.449456					
